# Enhancing Robustness of Variational Data Assimilation in Chaotic Systems: An α-4DVar Framework with Rényi Entropy and α-Generalized Gaussian Distributions

**DOI:** 10.3390/e27070763

**Published:** 2025-07-18

**Authors:** Yuchen Luo, Xiaoqun Cao, Kecheng Peng, Mengge Zhou, Yanan Guo

**Affiliations:** 1 College of Meteorology and Oceanology, National University of Defense Technology, Changsha 410073, China; luoyuchen23@nudt.edu.cn (Y.L.); zhoumengge23@nudt.edu.cn (M.Z.); 2 College of Computer Science, National University of Defense Technology, Changsha 410073, China; pengkc@nudt.edu.cn (K.P.); guoyn14@lzu.edu.cn (Y.G.)

**Keywords:** data assimilation, non-gaussian distribution, Rényi entropy, Lorenz-63 model, robustness

## Abstract

Traditional 4-dimensional variational data assimilation methods have limitations due to the Gaussian distribution assumption of observation errors, and the gradient of the objective functional is vulnerable to observation noise and outliers. To address these issues, this paper proposes a non-Gaussian nonlinear data assimilation method called α-4DVar, based on Rényi entropy and the α-generalized Gaussian distribution. By incorporating the heavy-tailed property of Rényi entropy, the objective function and its gradient suitable for non-Gaussian errors are derived, and numerical experiments are conducted using the Lorenz-63 model. Experiments are conducted with Gaussian and non-Gaussian errors as well as different initial guesses to compare the assimilation effects of traditional 4DVar and α-4DVar. The results show that α-4DVar performs as well as traditional method without observational errors. Its analysis field is closer to the truth, with RMSE rapidly dropping to a low level and remaining stable, particularly under non-Gaussian errors. Under different initial guesses, the RMSE of both the background and analysis fields decreases quickly and stabilizes. In conclusion, the α-4DVar method demonstrates significant advantages in handling non-Gaussian observational errors, robustness against noise, and adaptability to various observational conditions, thus offering a more reliable and effective solution for data assimilation.

## 1. Introduction

Data assimilation involves incorporating new observational data into numerical models during their dynamic operation. It takes into account the spatiotemporal distribution of the data, as well as the errors in the observational and background fields [1,2,3,4,5,6,7,8,9]. The mathematical basis of data assimilation lies in the fact that predicting a physical system requires both a model of the system’s time evolution and an estimate of its current state [10]. Within the dynamic framework of the model, data assimilation continuously combines observational information from different sources and resolutions, directly or indirectly, to automatically adjust the model. This process enhances the accuracy of the model state estimation and improves the model’s predictive capability.

Data assimilation methods can be categorized into two main types: sequential data assimilation methods and variational data assimilation methods [4]. Sequential methods, also known as single-time-level or kinematic methods, include optimal interpolation [11], successive correction [12], Kalman filtering [13], and particle filtering [14], among others. Variational methods, also referred to as multi-time-level or dynamic methods, primarily consist of 3-dimensional and 4-dimensional variational data assimilation methods (3D/4D-Var). The latter is an extension of the former in the time dimension and offers superior performance [15,16].

Since the 1990s, data assimilation has been successfully applied not only in atmospheric [5,17,18] and oceanic sciences [6,19,20] but also in various other fields such as Earth sciences [7,21], agricultural sciences [8,22,23], and artificial intelligence [9,24,25]. Among these, variational data assimilation transforms the processing of observational data into a functional minimization problem constrained by a dynamic model, which is an inverse problem [26]. By minimizing the objective function, it reduces the discrepancy between the analysis field and the observational data, thereby obtaining a more accurate initial field that is closer to the true state of the atmosphere or ocean. This helps improve the accuracy of numerical weather or ocean forecasting. For instance, Tanvir et al. (2016) [27] used a 3DVAR assimilation system to assimilate satellite radiance data, enhancing the short- to medium-term (approximately 60 h) forecast precision for Hurricane Sandy. Witold et al. (2019) [28] employed an improved 4DVar assimilation scheme to assimilate global navigation satellite system (GNSS) data, significantly improving humidity and precipitation forecasts, especially within the first 24 h, with a relative humidity error reduction of over 20% and more accurate precipitation forecasts. Shen et al. (2021) [29] used the 4.2 version of the Weather Research and Forecasting Data Assimilation (WRFDA) system to assimilate radar data, significantly enhancing the intensity, precipitation, and track prediction capabilities for Hurricane IKE. Wang et al. (2024) [30] combined the physical constraints of four-dimensional variational (4DVar) data assimilation technology with attention mechanism-based neural networks, achieving rapid and accurate estimation of multi-variable three-dimensional weather states. This significantly improved the efficiency and accuracy of medium- and long-term weather forecasting, providing strong support for the development of real-time weather forecasting systems.

However, in most data assimilation systems based on variational, ensemble, and hybrid methods, it is typically assumed that all minimized errors are Gaussian random variables [31,32,33]. In reality, observational data comes from different instruments with varying observation processes and principles, and the actual error distribution rarely meets the Gaussian distribution. For example, Chan et al. (2020) [34] noted that infrared radiometer brightness temperature data errors exhibit asymmetric distributions under complex weather conditions such as cloud cover and high aerosol concentrations. Hou et al. (2015) [35] pointed out that small precipitation events in automatic weather station precipitation observation data have discrete error distributions with a pronounced peak near zero. Traditional data assimilation methods struggle to effectively handle such complex error structures, leading to distorted assimilation results or even model divergence. Not all outliers are observations with severe errors. sometimes, observations without severe errors are classified as outliers because their random error distributions do not follow a Gaussian distribution [36]. The presence of outliers can significantly affect the accuracy of variational data assimilation analysis. Quality control can enable assimilation systems to effectively eliminate or absorb outliers, thereby obtaining a better analysis field. Given the potential impact of outliers on numerical prediction, developing new data assimilation algorithms that can robustly handle non-Gaussian errors and outliers has become an urgent need to enhance the accuracy and reliability of data assimilation.

In variational data assimilation optimization, the optimization of the objective function is often used to match computational data with observational data, thereby estimating physical model parameters. The least-squares objective function is widely used because it assumes that errors are independently and identically distributed according to a standard Gaussian probability distribution [37,38,39]. However, when errors exhibit non-Gaussian distribution, the least-squares estimate will produce deviations, violating the Gauss–Markov theorem [40,41]. In fact, a small number of outliers can render the least-squares criterion ineffective, thereby reducing the quality of the assimilation analysis.

In response to the shortcomings of traditional methods, this paper proposes a non-Gaussian and nonlinear data assimilation method called α-4DVar to address issues such as the unreasonable assumption of Gaussian-distributed observational errors in current variational data assimilation methods, the sensitivity of the objective functional gradient to observational noise and outliers, and the impact on analysis accuracy. This method primarily utilizes the heavy-tailed characteristics of Rényi entropy and the α-generalized Gaussian distribution to handle outliers. Comparative experiments with the traditional four-dimensional variational data assimilation method demonstrate that the α-4DVar method can effectively mitigate the impact of outliers on data assimilation, reduce sensitivity to observational errors, and enhance the robustness of data assimilation methods in the presence of strongly interfering observational data.

The remainder of this paper is organized as follows. Section 2 introduces Rényi entropy, the α-4DVar method derived from it, and the experimental configuration of the Lorenz-63 model. Section 3 presents the results, validating the strong robustness of the α-4DVar method through comparative experiments with the traditional 4DVar method. Finally, Section 4 provides a summary and discussion.

## 2. Methods

### 2.1. Rényi Entropy and the α-Generalized Gaussian Distribution

The concept of entropy originated within the domain of physics [42]. During his formulation of the quantitative expression for the second law of thermodynamics, the German physicist and mathematician Rudolf Julius Emanuel Clausius postulated the existence of a state function *S*, which he designated as entropy [43]. Subsequently, under the direct inspiration of Ludwig Boltzmann’s statistical entropy theory, Claude E. Shannon integrated Boltzmann’s entropy formalism into information theory in 1948. By employing statistical methodologies and the entropy equation, Shannon extended the thermodynamic concept of entropy into the field of communication, thereby establishing the foundational framework for information entropy [44]. Building upon this theoretical foundation, scientists rigorously defined the renowned Boltzmann–Gibbs entropy [45] (abbreviated as B-G entropy), as defined in Equation (1).

(1) $$S_{BG}(p)=-k\sum_{i=1}^{N}p(x_{i})\ln\left(p(x_{i})\right)$$

The system must satisfy the normalization condition and the unit variance equal to 1, that is

(2) $$\sum_{i=1}^{N}p(x_{i})=1$$

(3) $$\sum_{i=1}^{N}x_{i}^{2}p(x_{i})=1$$

**Figure 1:**
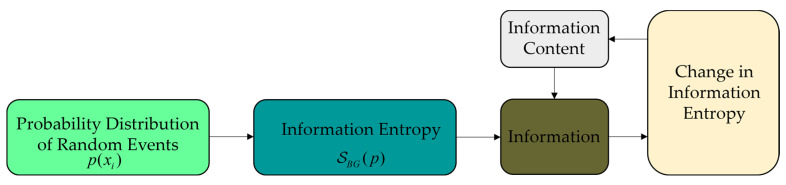
Interrelationships among probability, uncertainty, and information entropy.

In Equation (1), k represents the Boltzmann constant, $p(x_{i})(i=1,2,\ldots ,N)$ denotes the occurrence probability corresponding to signal $x_{i}$, and $p(x_{i})$ must satisfy the normalization condition. $x=y_{\mathrm{obs}}-y_{\mathrm{model}}=\{x_{1},x_{2},\ldots ,x_{N}\}$ represents the difference between the observed and model value. Here, N signifies all possible signals output by the source (i.e., the set of random events). Shannon indirectly quantified information content by measuring uncertainty, as illustrated in Figure 1.

Equation (1) is also referred to as information entropy or Shannon entropy. Typically, the Gaussian distribution can be derived from Shannon entropy under certain constraints [46]. In four-dimensional variational (4DVar) data assimilation, Gaussian assumptions for background error covariance and observation errors are conventional. However, actual error distributions often deviate from Gaussian characteristics. Recent advances in the physics community have proposed multiple forms of non-Shannon entropy [47], which offer broader adaptability. These diverse non-Shannon entropy formulations enable the derivation of novel error distribution models, thereby facilitating improvements to the objective function in data assimilation systems. Such enhancements allow the assimilation framework to address non-Gaussian error distributions or outliers more effectively.

Within the framework of information theory, Rényi [48] proposed the concept of α-entropy (Rényi entropy) in his seminal work, which serves as a single-parameter generalization of the conventional BGS entropy (i.e., Boltzmann–Gibbs–Shannon entropy). The Rényi entropy, also termed α-entropy, is defined in Equation (4).

(4) $$S_{\alpha }(p)=\frac{1}{1-\alpha }\ln\left(\sum_{i=1}^{N}p^{\alpha }(x_{i})\right)$$

where the exponent *α* distinguishes different entropy formulations. Compared to the BGS entropy (i.e., Equation (1)), the Rényi entropy (Equation (4)) shares several analogous properties, such as non-negativity, additivity, and attainment of its extremum under uniform probability distributions. However, a critical distinction between Rényi entropy and BGS entropy lies in the conservation property of entropy, which primarily depends on the selected exponent *α*.

Compared to the traditional Shannon entropy, Rényi entropy introduces a parameter *α*, offering greater flexibility in measuring different types of uncertainty. Different values of *α* correspond to distinct information measures, enabling a more nuanced characterization of a system’s diversity, uncertainty, and randomness.

When *α* < 1, the Rényi entropy exhibits greater sensitivity to low-probability events (rare events) and places heightened emphasis on the diversity or dispersion of the distribution.

When *α* > 1, it becomes more sensitive to high-probability events and prioritizes the concentration of dominant events within the distribution.

The Gaussian distribution can be derived by maximizing the Shannon entropy in conjunction with the likelihood function. Similarly, under specific constraints, maximizing the Rényi entropy leads to the derivation of an α-generalized Gaussian distribution, whose probability density function can be defined in Equation (5).

(5) $$p_{\alpha }(x)=A_{\alpha }{\left(1-\frac{\alpha -1}{3\alpha -1}x^{2}\right)}_{+}^{\frac{1}{\alpha -1}}$$

where the operator ${\left[x\right]}_{+}$ is defined as follows: when x<0, ${\left[x\right]}_{+}=0$, and when x≥0, ${\left[x\right]}_{+}=x$. This operation ensures that the functional expression evaluates to zero for negative x, thereby avoiding negative exponents—a requirement for validity in probability density functions.

$A_{\alpha }$ represents the normalization constant, given by

(6) $$A_{\alpha }=\left\{\begin{array}{l} \sqrt{\frac{1-\alpha }{[3\alpha -1]\pi }}\Gamma \left(\frac{1}{1-\alpha }\right)/\Gamma \left(\frac{1+\alpha }{2[1-\alpha ]}\right),\frac{1}{3}<\alpha <1\\ \sqrt{\frac{1-\alpha }{[3\alpha -1]\pi }}\Gamma \left(\frac{3\alpha -1}{2[1-\alpha ]}\right)/\Gamma \left(\frac{\alpha }{\alpha -1}\right),\alpha >1 \end{array}\right.$$

where $\Gamma \left(\cdot \right)$ represents the gamma function.

**Figure 2:**
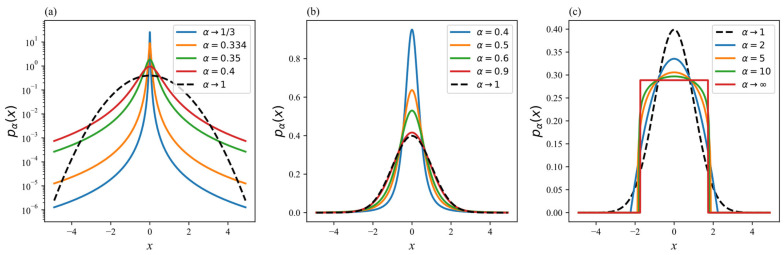
α-Gaussian probability distribution curves. The black dashed line represents the regular curve. (**a**) The curves of the probability density function under different *α* values: *α* → 1/3, *α* = 0.334, *α* = 0.35, and *α* = 0.4. (**b**) The curves of the probability density function under different *α* values: *α* = 0.4, *α* = 0.5, *α* = 0.6, and *α* = 0.9. (**c**) The curves of the probability density function under different *α* values: *α* = 2, *α* = 5, *α* = 10, and α → ∞.

Based on Equations (5) and (6), we plot representative curves of the α-generalized Gaussian distribution in Figure 2. Variations in the *α*-value significantly influence the shape of the distribution, particularly its tail thickness and peak height. As α→1/3, the probability distribution approaches a sharply peaked function. For α>1, increasing *α*-values lead to a reduction in the peak amplitude (maximum probability density) of the *α*-Gaussian distribution, resulting in a flatter profile. This implies that the central region of the distribution becomes more dispersed compared to the case of α=1 (standard Gaussian distribution). When α→1, the *α*-Gaussian distribution reduces to the standard Gaussian distribution. This arises because the Rényi entropy recovers the Shannon entropy as α→1, and maximizing Shannon entropy yields the Gaussian distribution. For $\frac{1}{3}<\alpha <1$, decreasing *α*-values within this range sharpen the peak of the α-Gaussian distribution while increasing its tail heaviness. Consequently, a larger proportion of the probability mass concentrates near the center, whereas the tail probabilities remain relatively small yet thicker than those of the standard Gaussian distribution. Such distributional characteristics render the α-Gaussian distribution particularly advantageous for modeling data exhibiting heavy-tailed behavior.

Heavy-tailed distributions offer enhanced capability to handle outliers and extreme values in data, avoiding the excessive sensitivity exhibited by light-tailed distributions toward such observations. Furthermore, they provide superior fitting to the heavy-tailed characteristics inherent in real-world datasets, thereby improving the accuracy and reliability of statistical models.

By adjusting the *α* parameter, the α-Gaussian distribution offers flexibility. It can characterize distributions ranging from the standard Gaussian to those with heavy-tailed characteristics. This adaptability endows the α-Gaussian distribution with broad application potential in numerical simulations and data assimilation methodologies. In data assimilation, datasets often contain outliers or extreme events, which can significantly impact parameter estimation and predictive outcomes of models. Heavy-tailed distributions exhibit superior robustness in handling noise and uncertainties, thereby enhancing model stability and reliability, which facilitates more stable parameter estimation and model fitting.

Building upon the application potential of the α-Gaussian distribution, we derive a 4D-Var-form objective function to replace the conventional one in 4DVar. Subsequently, we derive the gradient of the objective function and demonstrate its efficacy against outliers.

### 2.2. Non-Gaussian Nonlinear Data Assimilation Method Based on the α-Generalized Gaussian Distribution

The essence of the four-dimensional variational (4DVar) method lies in performing data assimilation within a fixed time window, fully utilizing observational information throughout this interval to ensure continuity in the assimilation process and obtain flow-dependent background error covariance. The continuous formulation of its objective function is given by [30]

(7) $$J(x_{0})=\frac{1}{2}{\left(x_{0}-x_{b}\right)}^{T}B^{-1}(x_{0}-x_{b})+\frac{1}{2}\int_{t_{0}}^{t_{N}}{\left(y_{t}-H(x_{t})\right)}^{T}R^{-1}\left(y_{t}-H(x_{t})\right)dt$$

where the term $\frac{1}{2}{\left(x_{0}-x_{b}\right)}^{T}B^{-1}(x_{0}-x_{b})$ represents the background term, where $x_{b}$ denotes the background state, $x_{0}$ is the initial state, and B is the background error covariance matrix. The observation term $\frac{1}{2}\int_{t_{0}}^{t_{N}}{\left(y_{t}-H(x_{t})\right)}^{T}R^{-1}\left(y_{t}-H(x_{t})\right)dt$ involves $y_{t}$, the observations at time *t*. $H(x_{t})$, the observation operator mapping model variables to observation space at time *t*. And R, the observation error covariance matrix. Here, $t_{0}$ and $t_{N}$ define the start and end times of the assimilation time window, respectively.

Under ideal conditions where both the background error and observation error follow Gaussian distributions, and with standardized processing of observational data, we can assume the observation error covariance matrix R to be the identity matrix, that is

(8) R=I

This is equivalent to transforming the observational data into a standard normal distribution space, where the error variance of each observation becomes unity. Consequently, the observation term in the objective functional simplifies to $\frac{1}{2}\int_{t_{0}}^{t_{N}}{\left\|y_{t}-H(x_{t})\right\|}^{2}dt$, and the objective functional can be expressed as Equation (9).

(9) $$J(x_{0})=\frac{1}{2}{\left(x_{0}-x_{b}\right)}^{T}B^{-1}(x_{0}-x_{b})+\frac{1}{2}\int_{t_{0}}^{t_{N}}{\left\|y_{t}-H(x_{t})\right\|}^{2}dt$$

For further simplification, $y_{t}-H(x_{t})$ can be expressed in scalar form as $y_{\mathrm{obs}}(t)-y_{\mathrm{model}}(t)$, where $y_{\mathrm{obs}}(t)$ denotes the observed value and $y_{\mathrm{model}}(t)$ represents the model-predicted value. The least-squares norm (L2-norm) formulation of the objective functional corresponds to the weighted sum of squares of the background term and observation term, that is

(10) $$\min J_{L_{2}}(x_{0})=\frac{1}{2}{\left(x_{0}-x_{b}\right)}^{T}B^{-1}(x_{0}-x_{b})+\frac{1}{2}\int_{t_{0}}^{t_{N}}\left[y_{\mathrm{obs}}(t)-y_{\mathrm{model}}(t)\right]dt$$

Assuming the observation term dominates in the objective functional and the background term’s influence is comparatively weakened, we neglect the background term for analytical simplicity and focus explicitly on the observation term within the objective functional, that is

(11) $$\min J_{L_{2}}(x_{0})=\frac{1}{2}{\int_{t_{0}}^{t_{N}}\left[y_{\mathrm{obs}}(t)-y_{\mathrm{model}}(t)\right]}^{2}dt$$

It is evident that Equation (11) represents the least-squares formulation of the objective functional, which quantifies the discrepancy between model-predicted values and observed values. The minimizer of this objective functional is equivalent to the maximum likelihood estimator of the joint probability density function under Gaussian-distributed background and observational errors. The gradient of the least-squares objective functional, i.e., the partial derivative with respect to the initial state component $x_{0i}$, is expressed by

(12) $$\frac{\partial J_{L_{2}}(x_{0})}{\partial x_{0i}}=-\int_{t_{0}}^{t_{N}}\frac{\partial y_{\mathrm{model}}(t)}{\partial x_{0i}}\left[y_{\mathrm{obs}}(t)-y_{\mathrm{model}}(t)\right]dt$$

Similar to Equation (11), the α-generalized Gaussian objective function [49] is given by

(13) $$\underset{x_{0}}{\min}J_{\alpha }(x_{0})=\frac{1}{1-\alpha }\int_{t_{o}}^{t_{N}}\ln\{1-\frac{\alpha -1}{3\alpha -1}{\left[y_{\mathrm{obs}}(t)-y_{\mathrm{model}}(t)\right]}^{2}\}dt$$

Next, we derive the partial derivative of the α-generalized Gaussian objective function $J_{\alpha }(x_{0})$ with respect to the initial state component $x_{0i}$ of the system. Let the integrand be defined in Equation (14).

(14) $$f(t)=\ln\left\{1-\frac{\alpha -1}{3\alpha -1}{\left[y_{\mathrm{obs}}(t)-y_{\mathrm{model}}(t)\right]}^{2}\right\}$$

So, Equation (13) can then be written as:

(15) $$\underset{x_{0}}{\min}J_{\alpha }(x_{0})=\frac{1}{1-\alpha }\int_{t_{0}}^{t_{N}}f(t)dt$$

To compute the partial derivative of Equation (15) with respect to $x_{0i}$, the order of integration and differentiation can be interchanged as follows:

(16) $$\frac{\partial J_{\alpha }}{\partial x_{0i}}=\frac{1}{1-\alpha }\int_{t_{0}}^{t_{N}}\frac{\partial f(t)}{\partial x_{0i}}dt$$

To compute $\frac{\partial f(t)}{\partial x_{0i}}$ in Equation (16), we apply the chain rule as follows:

(17) $$\frac{\partial f(t)}{\partial x_{0i}}=\frac{d}{dx_{0i}}\ln\left\{1-\frac{\alpha -1}{3\alpha -1}{\left[y_{\mathrm{obs}}(t)-y_{\mathrm{model}}(t)\right]}^{2}\right\}$$

This is equivalent to:

(18) $$\frac{\partial f(t)}{\partial x_{0i}}=\frac{1}{1-\frac{\alpha -1}{3\alpha -1}{\left[y_{\mathrm{obs}}(t)-y_{\mathrm{model}}(t)\right]}^{2}}\cdot \left(-\frac{\alpha -1}{3\alpha -1}\right)\cdot 2[y_{\mathrm{obs}}(t)-y_{\mathrm{model}}(t)]\cdot \frac{\partial [y_{\mathrm{obs}}(t)-y_{\mathrm{model}}(t)]}{\partial x_{0i}}$$

Simplifying Equation (18), that is

(19) $$\frac{\partial f(t)}{\partial x_{0i}}=-\frac{2(\alpha -1)}{\left(3\alpha -1)(1-\frac{\alpha -1}{3\alpha -1}{\left[y_{\mathrm{obs}}(t)-y_{\mathrm{model}}(t)\right]}^{2}\right)}[y_{\mathrm{obs}}(t)-y_{\mathrm{model}}(t)]\cdot \frac{\partial [y_{\mathrm{obs}}(t)-y_{\mathrm{model}}(t)]}{\partial x_{0i}}$$

In Equation (19), $y_{\mathrm{model}}(t)$, the model output, depends on the initial state $x_{0}$, whereas $y_{\mathrm{obs}}(t)$, the observed value, is independent of $x_{0}$. Therefore,

(20) $$\frac{\partial [y_{\mathrm{obs}}(t)-y_{\mathrm{model}}(t)]}{\partial x_{0i}}=-\frac{\partial y_{\mathrm{model}}(t)}{\partial x_{0i}}$$

Substituting Equation (20) into Equation (19), that is

(21) $$\frac{\partial f(t)}{\partial x_{0i}}=\frac{2(\alpha -1)}{\left(3\alpha -1)(1-\frac{\alpha -1}{3\alpha -1}{\left[y_{\mathrm{obs}}(t)-y_{\mathrm{model}}(t)\right]}^{2}\right)}[y_{\mathrm{obs}}(t)-y_{\mathrm{model}}(t)]\cdot \frac{\partial y_{\mathrm{model}}(t)}{\partial x_{0i}}$$

By substituting Equation (21) into Equation (16), that is

(22) $$\frac{\partial J_{\alpha }}{\partial x_{0i}}=\frac{1}{1-\alpha }\int_{t_{0}}^{t_{N}}\frac{2(\alpha -1)}{\left(3\alpha -1)(1-\frac{\alpha -1}{3\alpha -1}{\left[y_{\mathrm{obs}}(t)-y_{\mathrm{model}}(t)\right]}^{2}\right)}[y_{\mathrm{obs}}(t)-y_{\mathrm{model}}(t)]\cdot \frac{\partial y_{\mathrm{model}}(t)}{\partial x_{0i}}dt$$

By further simplifying Equation (22) and noting that $\frac{2(\alpha -1)}{1-\alpha }=-2$, that is

(23) $$\frac{\partial J_{\alpha }}{\partial x_{0i}}=-\frac{2}{3\alpha -1}\int_{t_{0}}^{t_{N}}\frac{\left[y_{\mathrm{obs}}(t)-y_{\mathrm{model}}(t)\right]}{1-\frac{\alpha -1}{3\alpha -1}{\left[y_{\mathrm{obs}}(t)-y_{\mathrm{model}}(t)\right]}^{2}}\cdot \frac{\partial y_{\mathrm{model}}(t)}{\partial x_{0i}}dt$$

Therefore, the gradient of the α-generalized Gaussian objective function is:

(24) $$\frac{\partial J_{\alpha }(x_{0})}{\partial x_{0i}}=-\int_{t_{0}}^{t_{N}}\{\frac{\partial y_{\mathrm{model}}(t)}{\partial x_{0i}}\frac{2[y_{\mathrm{obs}}(t)-y_{\mathrm{model}}(t)]}{3\alpha -1-(\alpha -1){\left[y_{\mathrm{obs}}(t)-y_{\mathrm{model}}(t)\right]}^{2}}\}dt$$

By comparing Equation (11) with Equation (24), the denominator term in Equation (24) $3\alpha -1-(\alpha -1){\left[y_{\mathrm{obs}}(t)-y_{\mathrm{model}}(t)\right]}^{2}$ varies with the residual $\left[y_{\mathrm{obs}}(t)-y_{\mathrm{model}}(t)\right]$. This enables adaptive adjustment of the gradient update step size based on the current model prediction accuracy. When the residual is large, the denominator increases, thereby reducing the gradient step size to avoid oscillations or divergence during optimization caused by excessively large steps. Conversely, when the residual is small, the denominator decreases, allowing larger gradient steps to accelerate convergence and improve optimization efficiency.

In Equation (11), the direct use of the squared difference may lead to excessively large gradient values when residuals are significant, compromising numerical stability. In contrast, Equation (24) introduces residual-dependent terms in the denominator to normalize the gradient. This normalization effectively mitigates numerical instability caused by large gradients, enhancing optimization stability, particularly for complex models or noisy data. The parameter *α* in Equation (24) provides flexible tuning to accommodate diverse data distributions and model characteristics. By adjusting *α*, the weights of terms in the denominator can be modified to meet optimization requirements under varying conditions. This flexibility allows Equation (24) to better handle practical challenges such as data heterogeneity and model complexity. Compared to the fixed formulation in Equation (11), Equation (24) exhibits superior generality and adaptability, achieving enhanced optimization performance across diverse applications while improving model robustness.

In the α-4DVar framework, the physical significance of parameter *α* is mainly reflected in characterizing error distributions and regulating the data assimilation process. The parameter *α* determines the tail thickness of the α-generalized Gaussian distribution. When 1/3 < *α* < 1, the distribution has a heavier tail, which better describes rare events with significant impacts. For instance, observational errors may exhibit heavy-tailed characteristics under extreme weather conditions. A smaller *α* value can more accurately capture such error distributions, thus enabling more precise handling of abnormal observational data in data assimilation. Meanwhile, parameter *α* can regulate the method’s sensitivity to outliers. When 1/3 < *α* < 1, the method is less sensitive to outliers, effectively dealing with data affected by noise. When *α* > 1, it becomes more sensitive to outliers, making it suitable for data with errors concentrated within a certain range. For example, in oceanography, for buoy observation data influenced by complex currents and local environments, selecting an appropriate *α* can optimize the assimilation effect.

### 2.3. Lorenz-63 Model

The Lorenz-63 model is a three-variable dynamical system proposed in 1963 by Edward Norton Lorenz, an American mathematician and meteorologist [50]. Derived from a simplification of the governing equations provided by Saltzman, this model has been widely employed to study atmospheric convection phenomena and chaotic behavior. The Lorenz-63 model is governed by the following three nonlinear ordinary differential equations [51], that is

(25) $$\left\{\begin{array}{l} \frac{dx}{dt}=\sigma \left(y-x\right)\\ \frac{dy}{dt}=x(r-z)-y\\ \frac{dz}{dt}=xy-bz \end{array}\right.$$

where x, y, and z are variables corresponding to the intensity of atmospheric convection, horizontal temperature variation, and vertical temperature variation, respectively. The constants σ, r, and b represent the Prandtl number, Rayleigh number, and aspect ratio, respectively. The standard parameter values are σ=10, r=28, and $b=\frac{8}{3}$.

**Figure 3:**
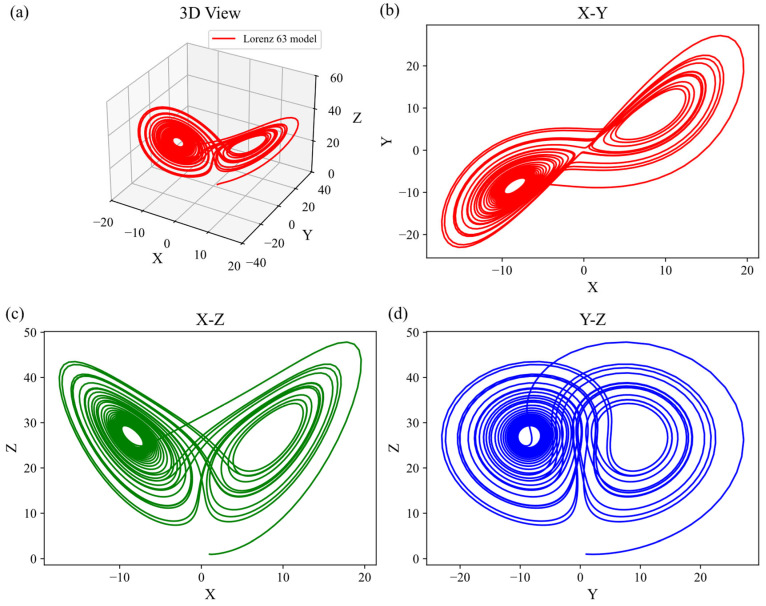
Lorenz-63 attractor: (**a**) 3D view, (**b**) X–Y 2D projection, (**c**) X–Z 2D projection and (**d**) Y–Z 2D projection.

Although the Lorenz-63 model is a simple three-variable system, it reveals the intrinsic nature of chaotic systems and the limitations of predictability. This model has been widely employed to validate various data assimilation algorithms and plays a pivotal role in studying the behavior and dynamical properties of chaotic systems. Assuming an initial guess value of $u_{s}=\left(x_{s},y_{s},z_{s}\right)$, even slight differences in the initial state can lead to entirely divergent trajectories—a phenomenon known as the butterfly effect. The solutions of the model form a structure resembling butterfly wings in phase space, hence termed the butterfly attractor. This structure has become an iconic representation of chaotic systems. As shown in Figure 3, the Lorenz-63 attractor with the initial value $u_{s}=\left(1,1,1\right)$ is illustrated.

In this study, we conduct numerical experiments using the Lorenz-63 model. The fourth-order Runge–Kutta method [52,53] is employed to solve the ordinary differential equations (ODEs), integrating the system from an initial state with a time step dt=0.01 to obtain the true state. Observations are then generated within an assimilation time window $L_{\mathrm{DA}}$ at a sampling interval $P_{\mathrm{DA}}$, with additive white noise of variance $\sigma_{\mathrm{obs}}^{2}$ introduced to simulate noisy observations. By varying the initial guess $u_{s}=\left(x_{s},y_{s},z_{s}\right)$, the assimilation window length $L_{\mathrm{DA}}$, and the sampling interval $P_{\mathrm{DA}}$, we compare the assimilation performance of the traditional 4DVar and the proposed α-4DVar under both noise-free and noisy observation conditions. Experimental conclusions are derived based on these comparisons.

## 3. Results

### 3.1. Comparative Experiments of Traditional 4DVar and α-4DVar Under Error-Free Observation Conditions in Lorenz-63

In this assimilation experiment, we set the number of integration steps to 2800, the assimilation window length to $L_{\mathrm{DA}}=5$, the sampling interval to $P_{\mathrm{DA}}=10$, the observation error variance to $\sigma_{\mathrm{obs}}^{2}=2$, the initial guess to $u_{s}=\left(2.0,3.0,4.0\right)$, and the *α* parameter to 0.9. The selection of *α* is determined based on the error characteristics.

**Figure 4:**
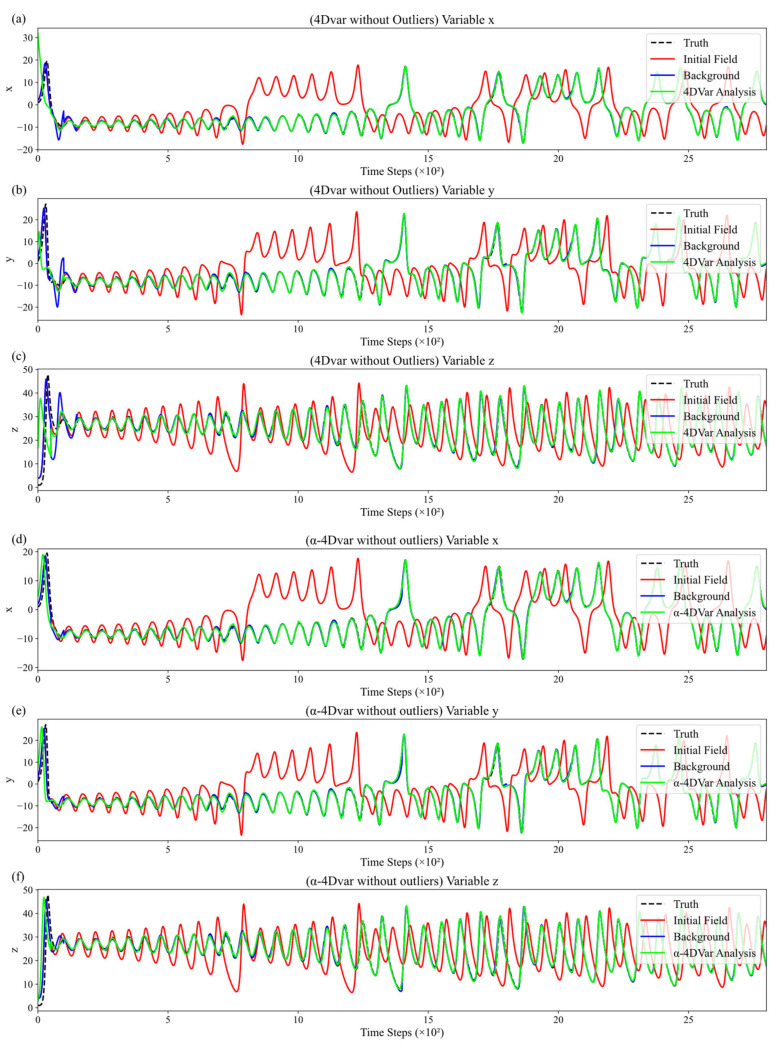
Temporal evolution of initial, background, and analysis fields for variables x, y, and z without outliers: (**a**–**c**) traditional 4DVar and (**d**–**f**) α-4DVar.

In this section, we conducted comparative experiments between the traditional 4DVar and the α-4DVar methods under a noise-free observation condition, as illustrated in Figure 4. Figure 4a–f show the variation in variable values with assimilation steps for the traditional 4DVar and α-4DVar methods, respectively. Each main plot is divided into subplots presenting the time series for the x, y, and z variables. The vertical axis denotes the variable values, whereas the horizontal axis represents the time steps, ranging from 0 to 2800.

In Figure 4, the black dashed line indicates the true variable values, the red solid line represents the initial field data, the green solid line shows the 4DVar analysis results, and the blue solid line denotes the background field. By analyzing the trends of the truth, initial field, background field, and analysis field in both assimilation methods, we observe that the analysis fields of both methods initially deviate slightly from the truth but quickly converge toward it, nearly overlapping with the truth curves. This demonstrates that, in the absence of observational errors, both data assimilation methods are similarly effective. They can efficiently correct the initial field by integrating observational data with model dynamic constraints, reducing the discrepancy between the initial field and the truth. Additionally, they rapidly capture the characteristics of truth changes, effectively utilizing observational information to adjust the model state and align it with the real system evolution. Furthermore, the comparable optimization outcomes achieved via different technical approaches confirm the robustness of the α-4DVar method, indicating that it performs on par with the traditional 4DVar method in the absence of observational errors.

**Figure 5:**
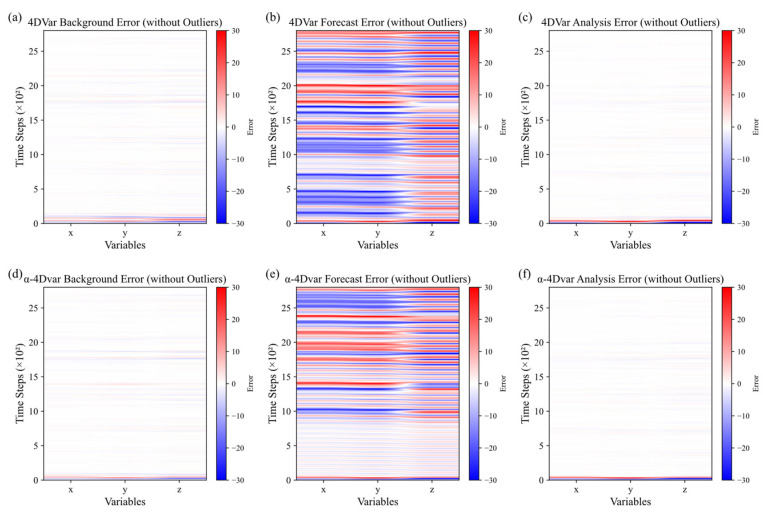
Heatmaps of background, forecast, and analysis errors without observational errors: (**a**–**c**) traditional 4DVar and (**d**–**f**) α-4DVar.

We also plotted heatmaps of background, forecast, and analysis errors for both the traditional 4DVar and α-4DVar methods in the absence of observational errors, as shown in Figure 5. Figure 5a–c are heatmaps of background, forecast, and analysis errors, respectively, for the traditional 4DVar method. Figure 5d–f are heatmaps of background, forecast, and analysis errors, respectively, for the α-4DVar method.

By comparing Figure 5a with Figure 5d and Figure 5c with Figure 5f, it is evident that both assimilation methods exhibit relatively small background and analysis errors under conditions without observational errors. The colors in these figures are close to the middle of the color spectrum, indicating stable error values with minimal fluctuations across time steps and variables.

When comparing Figure 5b with Figure 5e, the traditional 4DVar method shows significant fluctuations in forecast errors throughout the entire phase and across different variables. The color changes range from blue to red, indicating error variations between ±30. In contrast, the α-4DVar method demonstrates reduced forecast error fluctuations. The color changes are more constrained, with colors tending towards the middle of the spectrum and error values primarily fluctuating within ±10. The occurrence of extreme error values (close to ±30) is less frequent. Between time steps 0 and 1000, the error fluctuations are relatively smooth.

In the absence of observational errors, both methods show similar background and analysis errors, indicating stable and small errors. This suggests that under ideal conditions without observational errors, both methods can effectively control errors in the background and analysis fields. The primary difference between the two methods lies in their impact on forecast errors. The traditional 4DVar method exhibits larger forecast error fluctuations, with distinct regions of positive and negative errors, possibly due to its higher sensitivity to model errors and initial condition errors during the prediction process. Conversely, the α-4DVar method shows smaller forecast error fluctuations and a lower frequency of extreme error values, indicating its advantage in forecast error control and its ability to mitigate the impact of model and initial errors on prediction results, thereby enhancing prediction stability.

These experiments, conducted without observational errors, confirm that both methods can quickly converge the analysis field to the truth and correct initial field deviations, demonstrating the robustness of α-4DVar, which performs on par with traditional 4DVar. While both methods show comparable performance in background and analysis error control, the α-4DVar method outperforms the traditional method in forecast error control by reducing the influence of model and initial errors, thus improving prediction stability.

### 3.2. Comparative Experiments of Traditional 4DVar and α-4DVar Under Gaussian and Non-Gaussian Errors in Lorenz-63

Building on Section 3.1, we evaluated the methods using observational data with Gaussian and non-Gaussian errors. For Gaussian errors, outliers were dynamically set within [−3*σ*_obs_,+3*σ*_obs_], ensuring they fell within a reasonable error range and followed a zero-mean Gaussian distribution. These outliers constituted the Gaussian-error observational data. For non-Gaussian errors, outliers were generated using a Poisson distribution with *λ* = 3*σ*_obs_, and their signs were randomly chosen to avoid significant deviations from the normal error range. The resulting outliers exhibited non-Gaussian distribution characteristics and served as the non-Gaussian-error observational data.

**Figure 6:**
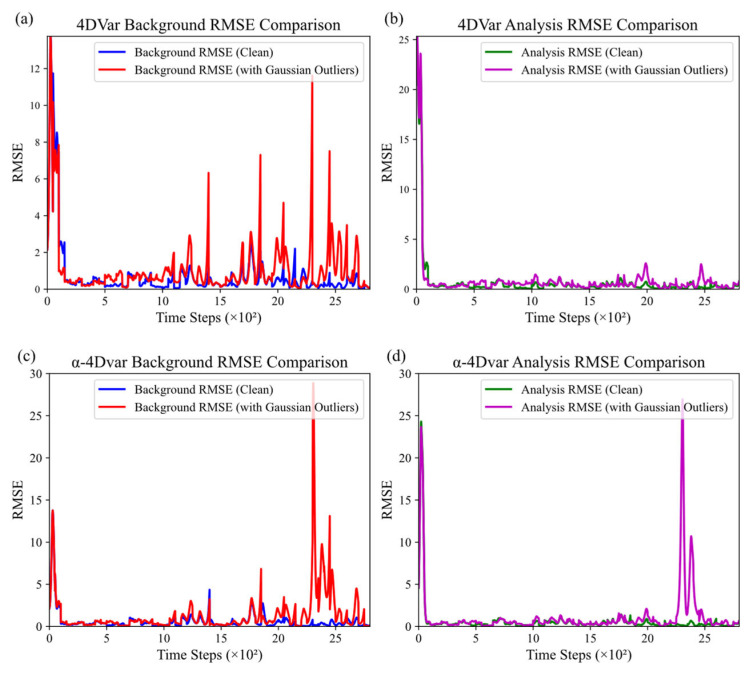
Comparison of background and analysis RMSE under Gaussian errors: (**a**,**b**) traditional 4DVar and (**c**,**d**) α-4DVar.

We plotted the background and analysis root mean square error (RMSE) variation curves of the traditional 4DVar and α-4DVar data assimilation methods with assimilation steps. RMSE measures the model-truth deviation. Figure 6a,b present the background and analysis RMSE curves for the traditional 4DVar method, while Figure 6c,d show the same for the α-4DVar method under observational data with 15% Gaussian errors. The horizontal axis represents time steps, and the vertical axis represents RMSE. The blue solid line indicates background RMSE without observational errors, the red solid line shows background RMSE with Gaussian observational errors, the green solid line denotes analysis RMSE without observational errors, and the purple solid line represents analysis RMSE with Gaussian observational errors.

As shown in Figure 6a, the background RMSE of the traditional 4DVar method starts high but quickly stabilizes in the absence of observational errors (blue line). This indicates effective background field adjustment towards the true state. However, with Gaussian observational errors (red line), the RMSE is higher and more volatile, indicating a detrimental impact on background field estimation. Figure 6b compares the analysis RMSE of the traditional 4DVar method with and without observational errors. Regardless of observational errors, the analysis RMSE decreases rapidly and remains low (green and purple lines), demonstrating effective data integration for accurate analysis fields.

In Figure 6c, the α-4DVar method shows similar background RMSE behavior to the traditional method. Without observational errors, the RMSE decreases and stabilizes. With Gaussian errors, the RMSE is lower than the traditional method’s from steps 100 to 2000 but shows a peak at step 2300, stabilizing by step 2500. For the α-4DVar analysis RMSE (Figure 6d), it quickly decreases and stabilizes without observational errors (green line). With Gaussian errors (purple line), it remains low and stable after the initial drop, though with a temporary peak between steps 2300 and 2500.

In summary, the traditional 4DVar and α-4DVar data assimilation methods perform similarly in the absence of observational errors. When Gaussian observational errors are present, α-4DVar, while slightly less effective than traditional 4DVar, still achieves comparable results.

**Figure 7:**
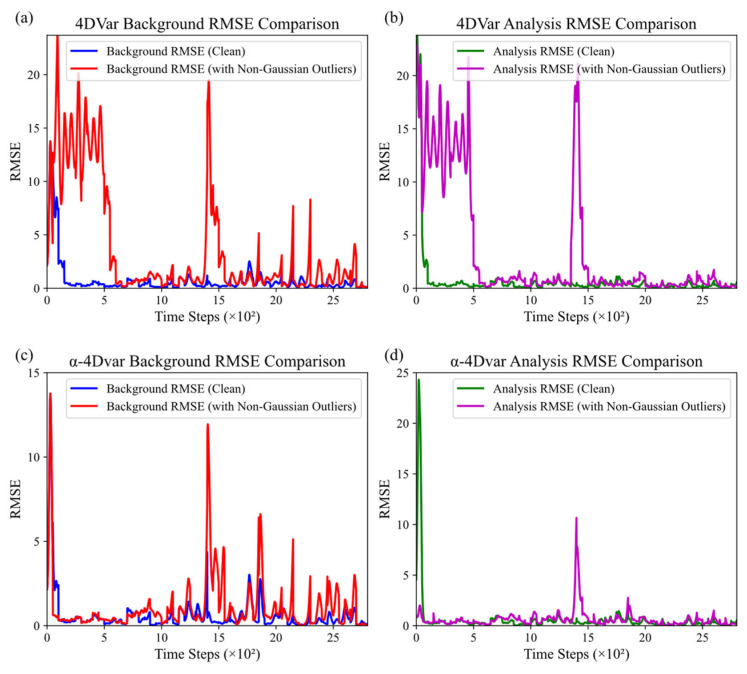
Comparison of background and analysis RMSE under non-Gaussian errors: (**a**,**b**) traditional 4DVar and (**c**,**d**) α-4Dvar.

Subsequently, under the scenario of observational data containing 15% non-Gaussian errors, we plotted the variation curves of background and analysis root mean square errors (RMSEs) of the traditional 4DVar and α-4DVar data assimilation methods with assimilation steps, as shown in Figure 7.

As shown in Figure 7a,c, the background RMSE of the 4DVar method increases and fluctuates significantly under non-Gaussian observational errors, with notable peaks within time steps 0–500. This indicates that non-Gaussian errors substantially interfere with the background field assimilation of 4DVar, increasing and destabilizing the background field error. In contrast, the α-4DVar method shows reduced background RMSE, smaller fluctuation, and lower peak amplitudes, demonstrating its ability to mitigate the impact of non-Gaussian errors and enhance background field accuracy.

Figure 7b,d reveal that the analysis RMSE of the 4DVar method also increases and fluctuates under non-Gaussian errors, especially within time steps 0–500. Compared to 4DVar, the α-4DVar method achieves significantly improved analysis RMSE, with smaller error values and milder fluctuations. This indicates that α-4DVar can generate more reliable analysis fields and shows stronger robustness against non-Gaussian observational errors.

In summary, α-4DVar outperforms traditional 4DVar in assimilating data with non-Gaussian errors, particularly in analysis field assimilation. It can better resist the interference of non-Gaussian observational errors, providing more reliable initial conditions for subsequent numerical forecasting or state estimation.

To quantify the computational overhead of α-4DVar, we compared the total runtime for completing 2800 assimilation steps between traditional 4DVar and α-4DVar (α = 0.9) in the same hardware environment, based on the two experiments in Section 3.2. Timing covered the entire process from initial field loading to analysis field output, including objective function calculation, gradient solving, and optimization iterations. Each experiment was repeated 10 times, with the average taken to eliminate system fluctuation effects. Consumed Time (G) denotes the total time consumed under Gaussian error conditions, while Consumed Time (NG) refers to that under non-Gaussian error conditions.

As shown in Table 1, traditional 4DVar took 16.83 s and 17.96 s under Gaussian and non-Gaussian errors, respectively. In comparison, α-4DVar took 20.04 s (a ~20% increase) under Gaussian errors and 23.45 s (a ~30% increase) under non-Gaussian errors. Thus, α-4DVar had longer runtimes in both error conditions. This is mainly because α-4DVar handles non-Gaussian errors and leverages Rényi entropy and α-generalized Gaussian distribution characteristics, making it more complex.

Traditional 4DVar and α-4DVar only differ in gradient iteration during computation. Despite its higher computational cost, α-4DVar offers significant practical advantages in handling non-Gaussian errors and outliers, providing more reliable analysis results and justifying the extra cost.

### 3.3. Comparative Experiments of α-4DVar with Different Initial Guesses in Lorenz-63

Due to the sensitivity of the Lorenz-63 model, where even minor differences in initial conditions can lead to entirely divergent state evolutions, we conducted comparative experiments with different initial guesses.

Building on the settings described in Section 3.1, we defined the initial guesses as $u_{s1}=\left(2.0,3.0,4.0\right)$, $u_{s2}=\left(2.2,3.2,4.2\right)$, $u_{s3}=\left(2.5,3.5,4.5\right)$ and $u_{s4}=\left(3.0,4.0,5.0\right)$, while keeping other configurations unchanged. Experiments were carried out with 15% non-Gaussian observational errors.

**Figure 8:**
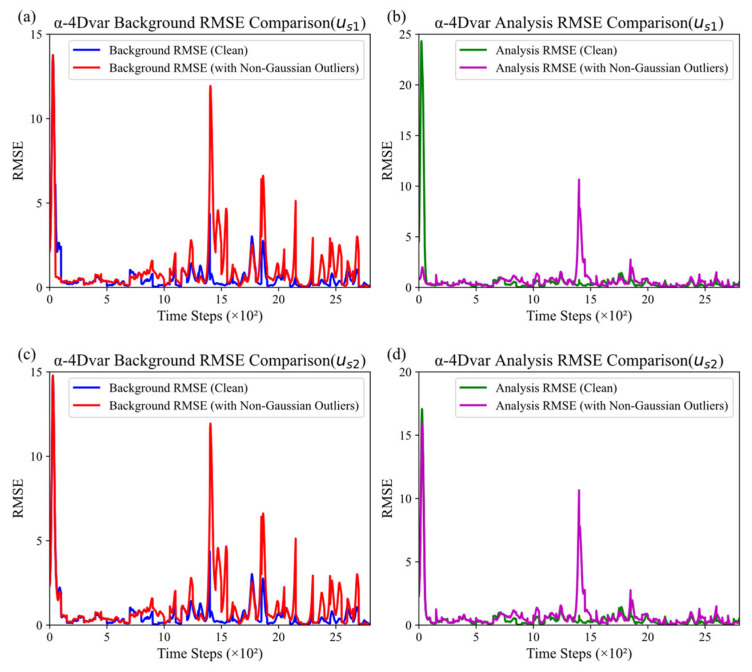

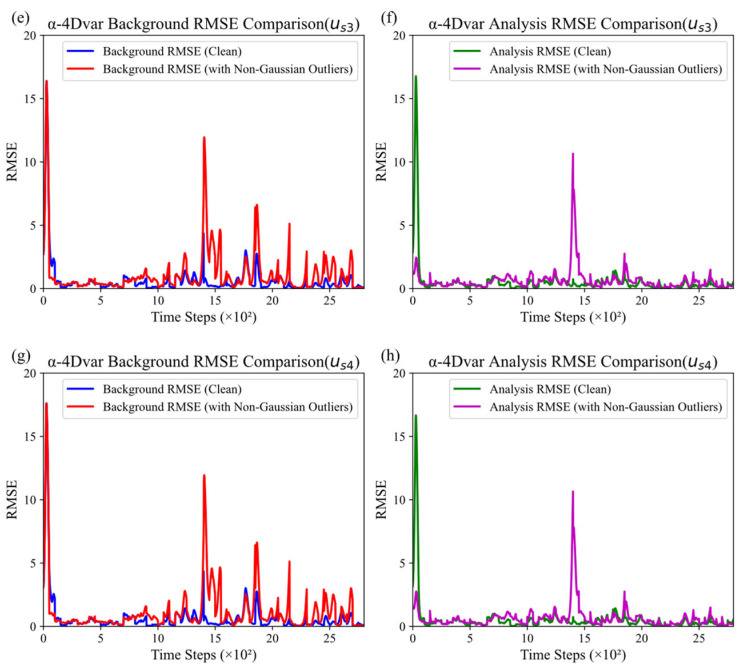
Comparison of background and analysis RMSE for the α-4DVar under different initial guesses: (**a**,**b**) *u*_s1_, (**c**,**d**) *u*_s2_, (**e**,**f**) *u*_s3_, and (**g**,**h**) *u*_s4_.

We plotted the background and analysis RMSE variation curves of the α-4DVar method with different initial guesses, as shown in Figure 8. Subplots (a) and (b), (c) and (d), (e) and (f), and (g) and (h) in Figure 8 correspond to the background and analysis RMSE curves for *u*_s1_, *u*_s2_, *u_s_*_3_, and *u*_s4_, respectively.

In Figure 8, for different initial guesses, both the background and analysis root mean square error (RMSE) show similar trends. Initially, differences exist in both background and analysis RMSE due to varying initial guesses. As the initial guess increases, the initial background RMSE peak becomes larger in the non-Gaussian error case, while the initial analysis RMSE peak decreases in the error-free case. Overall, as time steps increase, despite minor fluctuations in the middle and late stages, both background and analysis RMSE show a general downward trend toward stability. This indicates that the α-4Dvar method can optimize the background and analysis fields over time, reducing errors and making them converge to a stable level. This demonstrates the method’s adaptability and optimization capability in data assimilation.

In summary, under different initial guesses, the α-4DVar method shows similar trends in background and analysis RMSE. It demonstrates adaptability and robustness, effectively lowering RMSE over time, even with initial deviations from the truth.

## 4. Conclusions and Discussion

This paper introduces a non-Gaussian and nonlinear variational data assimilation method called α-4DVar, based on Rényi entropy and the α-generalized Gaussian distribution (GGD). It aims to tackle the problem of the unrealistic Gaussian observation error assumption and the sensitivity to observation noise and outliers in traditional variational data assimilation methods. A series of numerical experiments on the Lorenz-63 model were conducted to evaluate the α-4DVar method under various observation conditions and to compare it with the traditional 4DVar method. The discussion is organized around three aspects: experimental results analysis, the method’s advantages and limitations, and future research directions.

The experimental results show that the α-4DVar method performs comparably to the traditional 4DVar method in the absence of observation errors, confirming its robustness and ability to effectively correct initial field deviations and quickly approach the truth under ideal conditions. This outcome aligns with previous research experience and indicates that the α-4DVar method can yield good assimilation results when observation errors are controllable. However, in practical applications, observation errors are inevitable, and non-Gaussian errors can significantly impact assimilation results.

Under Gaussian error conditions, although the α-4DVar method initially underperforms slightly compared to the traditional 4DVar method, it eventually achieves comparable results. This suggests that the α-4DVar method can adapt to Gaussian error interference to some extent but requires further optimization to enhance its robustness under Gaussian error conditions. In contrast, under non-Gaussian error conditions, the advantages of the α-4DVar method become evident. It significantly reduces root-mean-square error (RMSE) in both the background and analysis fields, demonstrating stronger anti-interference and stability. This result is consistent with previous findings that traditional methods perform poorly under non-Gaussian error conditions. Our study further shows that introducing the characteristics of the α-GGD and Rényi entropy can effectively improve the performance of data assimilation methods in non-Gaussian error environments.

We also examined the impact of different initial guess values on the α-4DVar method. The results indicate that regardless of the initial guess value, the α-4DVar method can gradually reduce RMSE in both the background and analysis fields and stabilize. This demonstrates the method’s adaptability and robustness to initial conditions, enabling it to obtain reliable assimilation results even when the initial guess value deviates from the truth.

The α-4DVar method offers significant advantages in handling non-Gaussian observation errors, anti-interference capabilities, and adaptability to different observation conditions, providing a more reliable and effective solution for data assimilation. However, the method has limitations, such as increased computational complexity, dependence on the parameter, and slightly weaker performance under Gaussian error conditions. In addition, the choice of α has some impact on the performance. While this study experimentally confirmed its effectiveness across various scenarios, further research is still needed to optimize *α* selection for practical applications. Future research should focus on optimizing and improving the α-4DVar method to overcome these limitations and enhance its performance and reliability in practical applications.

From a broader perspective, this study provides a new approach and methodology for the field of data assimilation. The limitations of traditional 4DVar methods in handling non-Gaussian errors and outliers have long been a pressing issue. The successful application of the α-4DVar method offers a feasible solution to this problem and provides new research directions. Future research could explore the application of the α-4DVar method in more complex models and real observation data, such as high-resolution numerical models in atmospheric and ocean sciences, to verify its effectiveness and feasibility in practical scenarios. Further research on the selection and optimization of the value of *α* is needed to establish a more scientific and reasonable method for determining the value of *α* based on data distribution characteristics in different scenarios.

Additionally, combining the α-4DVar method with other advanced data assimilation techniques, such as ensemble Kalman filters and particle filters, could leverage their respective advantages to improve the accuracy and efficiency of data assimilation. Finally, for specific types of observation errors or complex data distributions, exploring ways to improve the α-4DVar method or developing new non-Gaussian data assimilation algorithms could meet the needs of different application fields.

## Figures and Tables

**Table 1:** The time consumed by the 4DVar and α-4DVar.

Method	Assimilation Steps	Consumed Time(G)	Consumed Time(NG)
4DVar	2800	16.83 s	17.96 s
α-4DVar (*α* = 0.9)	2800	20.04 s	23.45 s
Time Increment	\	19.1%	30.5%

## Data Availability

The original contributions presented in this study are included in the article. Further inquiries can be directed to the corresponding authors.

## Abbreviations

The following abbreviations are used in this manuscript:

4DVar	Four-dimensional variational data assimilation
RMSE	Root mean square error
GNSS	Global navigation satellite system
WRFDA	Weather Research and Forecasting Data Assimilation
BGS	Boltzmann–Gibbs–Shannon entropy
ODEs	Ordinary differential equations
GGD	Generalized Gaussian distribution
